# Duration of surgical antibiotic prophylaxis and surgical site infection in orthopaedic surgery: a prospective cohort study

**DOI:** 10.1097/JS9.0000000000001881

**Published:** 2024-07-17

**Authors:** Zhenbang Yang, Yuchuan Wang, Zhongzheng Wang, Junyong Li, Pei Du, Hongyu Meng, Kuo Zhao, Junzhe Zhang, Ming Li, Zhucheng Jin, Ziheng Peng, Dandan Ye, Kai Ding, Hongzhi Lv, Juan Wang, Xin Xing, Zhaohui Song, Wei Chen, Yanbin Zhu, Yingze Zhang

**Affiliations:** a Department of Orthopedic Surgery, The Third Hospital of Hebei Medical University, Shijiazhuang; b Department of 1st Foot and Ankle Surgery, Cangzhou Integrated Traditional Chinese and Western Medicine Hospital, Cangzhou City; c Department of Orthopedic, Wuxi Hand Surgery Hospital, Wuxi, Jiangsu; d Department of Gastroenterology, Xiangya Hospital Central South University, Changsha, Hunan, People’s Republic of China

**Keywords:** antibiotic prophylaxis, duration, orthopaedic surgery, prospective study, surgical site infection

## Abstract

**Background:**

The optimal duration for surgical antibiotic prophylaxis (SAP) for preventing surgical site infection (SSI) in orthopaedic surgeries remains poorly supported by high-level evidence. This study aimed to assess the association between SAP duration and the occurrence of SSI within one year postoperatively.

**Methods:**

This prospective cohort study was based on the database from Surgical Site Infection Surveillance and Improvement Project (SISIP) of a tertiary orthopaedic university hospital from October 2014 to December 2020. The main outcome was SSI, defined according to the CDC/NHSN criteria, determined by a review of index hospitalization medical records, microbiology laboratory reports, and readmission records for SSI treatment within one year after discharge. Adjusted generalized additive models (GAMs) were performed to assess the relationships between SAP duration and SSI, determine the cut-off point of SAP duration, and estimate the relative contribution of each included variable across the overall cohort and the three subgroups (open fracture, closed fracture, and non-traumatic group). Multivariable logistic regression models were used to estimate the association between prolonging SAP duration based on the cut-off point and SSI.

**Results:**

There were 37 046 patients (55.1% male) included, with an overall SSI incidence of 2.35% (871/37 046). In adjusted GAMs, no statistically significant relationships were observed in the overall cohort and open or closed group (*P*>0.05), but a non-linear relationship was exhibited in the non-traumatic group (*P*=0.03); the cut-off point was 2.4 days for the overall cohort and 3.6 days (open), 2.6 days (closed), 1.1 days (non-trauma) for three subgroups. In adjusted logistic regression, prolonging SAP duration did not demonstrate a statistically significant protective effect in overall cohort (aOR=0.868; 95% CI, 0.746–1.011) and three groups (open: aOR=0.867; 95% CI, 0.668–1.124; closed: aOR=0.925; 95% CI, 0.754–1.135; non-trauma: aOR=1.184; 95% CI, 0.832–1.683). The relative contribution ranks of SAP duration were 21st overall among 34 factors, 14th for open fractures, 28th for closed fractures, and 3rd for the non-traumatic group among 33 factors.

**Conclusion:**

Prolonged postoperative SAP duration has no protective effect against SSI in orthopaedic surgery. Our findings support current guidelines against the practice of continuing SAP postoperatively.

## Introduction

Highlights

- A prospective cohort study with large sample size, extensive data collection, and adjustment for potential confounders to elucidate the relationship between the duration of surgical antibiotic prophylaxis and surgical site infection in Chinese patients with fracture.
- Quantifying the relative contribution of the duration of surgical antibiotic prophylaxis among 34 potential factors affecting surgical site infection helps healthcare providers assess the priority of this intervention and make informed decisions regarding antibiotic administration.

Surgical site infection (SSI) is the most common healthcare-associated infection worldwide, causing substantial morbidity and mortality^1^. Surgical antibiotic prophylaxis (SAP) has long been recognized as a routine measure for SSI prevention^2^. However, whether continuing SAP after surgery can further reduce SSI is unclear. Increasing evidence shows that prolonged SAP is associated with the emergence of antibiotic resistance and adverse events (e.g. toxicity, allergic reactions, acute kidney injury)^3^. Despite this, prolonged SAP (i.e. >1 day) after surgery remains a common practice in all regions, with reported percentages of patients receiving it ranging from 29.5 to 92.5%^4^.

Orthopaedics is the fourth most common type of surgery worldwide^5^. With the goal of optimizing antibiotic stewardship (i.e. appropriate antibiotic choice, indications, duration, and dosage)^6^, a critical approach aimed at combating resistance, reducing adverse effects, and ensuring the most effective treatment, six guidelines have been developed in the past two decades^2,7–11^. However, their recommendations for SAP duration vary, ranging from single infusions to durations up to 3 days, primarily relying on a relatively low-level evidence base: six meta-analyses^12,13^ and outdated reviews^14–17^ with moderate certainty, 7 RCTs but all published before 2008^18–24^, and even a retrospective observational study^23^. Additionally, orthopaedics encompasses multiple subspecialities (trauma, spinal, arthroplasty, etc.) involving surgical procedures, for example, debridement, arthroscopy, internal fixation, and artificial joint prosthesis; thus, SSI rates vary greatly, ranging from 0.2 to 51.8%^10,11^, potentially making these guideline recommendations less pertinent and applicable in practice. Moreover, these studies are often underpowered (sample size, 140–635) to detect between-group differences in SSIs that are so rare in practice and also fail to standardize or adjust for important confounders or covariates related to the antibiotic stewardship (e.g. antibiotic choice, preoperative and postoperative dosage), making it difficult to derive conclusive evidence on the true association between SAP duration and SSI. In 2019, an international questionnaire among members registered with AO Trauma showed that the majority (71% out of 1197) of orthopaedic surgeons agreed with the statement ‘the optimal duration of systemic perioperative antibiotic prophylaxis in the treatment of open fractures has not been well defined in the literature’^25^. On the other hand, despite the fact that a variety of factors (e.g. demographic data, antibiotic strategies, medical comorbidities, surgery data, and laboratory indicators) have been well established as risks or mitigation for SSI over the past decades^26,27^, the relative contribution (ranking) of SAP duration among these factors is unknown^28^. This may lead clinicians to regard antibiotics as a panacea, for example, arbitrarily prolonging SAP in cases involving a higher risk of SSI (e.g. open fractures, contaminated wounds, elderly, undernourished, or immunocompromised individuals). Consequently, this is also a considerable source of antibiotic overuse.

Thus, the aim of our study was to assess the relationship between SAP duration and the incidence of SSI in orthopaedic surgery, and to quantify the relative contribution of SAP duration among the common factors associated with SSI occurrence.

## Method

### Data source

Data were obtained from the Surgical Site Infection in Orthopaedic Surgery (SSIOS) database, a prospectively manually maintained and annually updated database of inpatients who underwent orthopaedic surgeries at our hospital since October 2014. The database was part of the Surgical Site Infection Surveillance and Improvement Project (SISIP) in our institution, which is a university-affiliated tertiary referral teaching hospital in China’s central province (with over 75.9 million population of the jurisdiction). The SSIOS database was established by a leadership team consisting of 15 experienced professionals, including orthopaedic surgeons, radiologists, laboratory, infection control specialists, and senior nurses. A total of 60 interns underwent a one-month training led by a leadership team, randomly assigned to three groups, each responsible for collecting specific information: (1) management and clinical data from electronic medical records (Kaihua Network Technology Co., Ltd., Beijing); (2) imaging examination data from Picture Archiving and Communication System (iMedical PACS, DHC Software Co., Ltd., Beijing), and (3) laboratory and bacterial culture results from RuiMei Laboratory Information System (RMLIS, Rui Mei Computer Technology Co., Ltd., Shanghai). Trained clinical nurses with more than 3 years of experience, together with experienced orthopaedic surgeons, radiologists, and laboratory doctors, formed the follow-up team. Follow-up was conducted through telephone, WeChat (Tencent Holdings Ltd., Shenzhen, China), and outpatient visits at 1, 6, and 12 months after discharge. Patients who have not responded will be contacted again after a 24-h interval, and if there is still no response, they will be considered lost to follow-up. Double data entry and validation minimized data entry errors using EpiData software (3.1 for Windows, EpiData Association, Odense, Denmark).

### Study cohort and study design

The study population included 47 837 adult patients (≥18years old) enrolled in the SSIOS database between October 2014 and December 2020. Patients who met the following criteria were excluded: (1) special types of fractures: old fractures, pathological fractures, or patients combined with tumours; (2) therapeutic use of antibiotics: long-term use of antibiotics postoperatively (more than a week), preoperative infection, or special infection; (3) no antibiotic prophylaxis perioperative, or non-cephalosporin antibiotic prophylaxis; and (4) lost to follow-up. Orthopaedic surgeries are commonly distinguished as three subspecialties based on varying SSI risks and antibiotic regimens: open fractures surgeries, closed fractures surgeries, and non-traumatic surgeries. Consequently, the overall cohort was divided into three subgroups: open fracture, closed fracture, and non-traumatic group. The present study adhered to the principles outlined in the Helsinki Declaration, and the findings have been reported following the STROCSS criteria^29^ (Supplemental Digital Content 1, http://links.lww.com/JS9/C942). Because the clinical data involved in this article belonged to the secondary use, the requirement of informed consent for the use of patient data was exempted by the local ethics committee.

### Exposure variable

The main exposure variable was the SAP duration (in days) used perioperatively, determined through double data retrieval from clinical courses and physician orders of medical records. Other antibiotic regimen relevant information simultaneously recorded included the choice of antibiotic, as well as preoperative and postoperative dosage (Supplementary Table S1, Supplemental Digital Content 2, http://links.lww.com/JS9/C94 https://1drv.ms/w/s!AvMSQa964oY8iEVlkaY3rhc29WHg?e=yqTQkB). Each patient received intravenous antibiotic prophylaxis 0.5–1 h before surgery, and the same antibiotic choice was administered postoperatively.

### Covariates

Based on risk factors for SSI occurrence identified in previous literature and our experience, we considered four categories of covariates: demographic information, medical comorbidities, surgery data, and laboratory indicators. Demographic information included age, sex, place of residence, and lifestyle factors (smoking status, alcohol consumption, and body mass index). Medical comorbidities included hypertension, diabetes mellitus (DM), cerebrovascular disease (CVD), coronary heart disease (CHD), chronic kidney disease (CKD), chronic liver disease (CLD), chronic obstructive pulmonary disease (COPD), peripheral vascular disease (PVD), surgical history, and American Society of Anaesthesiologists (ASA) score. Laboratory indicators included white blood cell (WBC), red blood cell (RBC), albumin (ALB), serum creatinine (CREA), and activated partial thromboplastin time (APTT). Surgery data included wound class, surgery duration (in hours), year, type (replacement, implant, debridement, osteotomy, arthroscopy, or bone grafting), location (spine, shoulder, upper, elbow, forearm, hand, pelvis, knee, calf, hip, thigh, ankle, or a combination of the above), emergency (yes or no), anaesthesia method (general anaesthesia or other), cause of surgery, and type of blood transfusion (none, autologous, allogeneic or both autologous and allogeneic). Additionally, we also factored in steroid use (yes or no) and choice and dosage of SAP as covariates.

### Outcomes

Our primary outcome was the incidence of SSI. SSI was defined according to the criteria of the Centers for Disease Control and Prevention (CDC) National Healthcare Safety Network (NHSN)^30^, diagnosed by infection control physicians based on microbiological tests for inpatients, and confirmed by readmission records or qualified microbiology laboratory cultures results during the one-year follow-up of discharged patients. The identical results of the culture and drug susceptibility test (DST) obtained from isolates collected from the same patient during the same hospitalization are counted only once. The time of occurrence (during hospitalization or after discharge) and strains of SSI were simultaneously recorded for subsequent sensitivity and exploratory analyses further to evaluate the relationship between SAP duration and SSI. Multidrug-resistant (MDR) bacterial strains were shown in the Supplementary Methods, Supplemental Digital Content 2, http://links.lww.com/JS9/C94 (https://1drv.ms/w/s!AvMSQa964oY8iEVlkaY3rhc29WHg?e=yqTQkB).

### Statistical analysis

Categorical covariables were expressed as numbers (%) and analyzed using the *χ* ^2^ test. Continuous covariables were expressed as mean±standard deviation (SD) or median [Q1–Q3] depending on the normality and compared by one-way ANOVA analysis of variance or Kruskal–Wallis *H* test, as appropriate. Missing data were handled through the single predictive mean matching (PMM) imputation based on other patient characteristics (Supplementary Table S10, Supplemental Digital Content 2, http://links.lww.com/JS9/C94)^31^. Prior to any regression analysis and modeling, we tested multicollinearity among all variables by checking for the variance inflation factor (VIF), and a VIF of greater than 2 was considered multicollinearity.

The adjusted generalized additive models (GAMs) were used to model the risk of SSI according to SAP duration (in days) and estimate the relative contribution of each included variable. The optimal cut-off value for SAP duration was determined by considering the intersection point between log (odds ratio) and the *x*-axis and existing data from literature reports^11,12^, and accordingly patients were dichotomized. To mitigate overfitting, the least absolute shrinkage and selection operator (LASSO) regression was used to select variables for adjustment^32^. In adjusted GAMs, continuous variables were fitted by penalized thin plate regression splines and categorical variates were treated as factors; the maximum likelihood (ML) method was employed, with the model framework specified as *Poisson* regression^33^. The relative contribution of each variable was calculated using the variance (*χ* ^2^) minus its *dƒ* (estimated for spline terms)^28,34^, with a higher value indicating a greater contribution.

Multivariable logistic regression models were used to assess the association between the SAP duration (GAM-determined dichotomous variable) and the incidence of SSI across the overall cohort and three subgroups, adjusting for variables with a univariate *P* value <0.20. Potential population heterogeneity was determined by testing the interaction between SAP duration and the following terms: age, sex, BMI, and CREA^3^.

Several sensitivity analyses were performed as follows: (1) excluding SSIs acquired outside the hospital; (2) including medical payment type as further covariables^35^; (3) excluding surgeries performed on holidays and weekends^36^; (4) excluding surgeries performed with non-chief surgeons^37^; (5) excluding patients receiving multi-surgeries for the index hospitalization; (6) conducting complete data analysis; and (7) conducting stratified analyses by GA (Gustilo–Anderson) grade in for open fracture group^38^. Also, to explore whether the WHO guidelines for SSI prevention have an impact on the relationship between SAP duration and SSI, we conducted a stratified analysis by dividing the overall cohort into the prior (before January 2017) and latter (after December 2017) group based on the year of guideline publication (December 2016). Furthermore, several exploratory analyses were conducted using logistic regression analyses adjusting for the same covariables as the main model as follows: (1) considering SAP duration as an ordered categorical variable, to examine the duration-dependent relationship between SAP duration and SSI in open and closed fracture groups; (2) treating the incidence of methicillin-resistant *Staphylococcus aureus* (MRSA), MDR, and multiple infections as dependent variables, respectively^3^.

All analyses were performed with R software 4.1.3 (R Foundation for Statistical Computing, Vienna, Austria) and SPSS 26.0 (IBM Corp, Armonk, New York, USA), and *P*<0.05 indicates the statistical significance of the difference.

## Results

### Baseline characteristics

**Figure 1:**
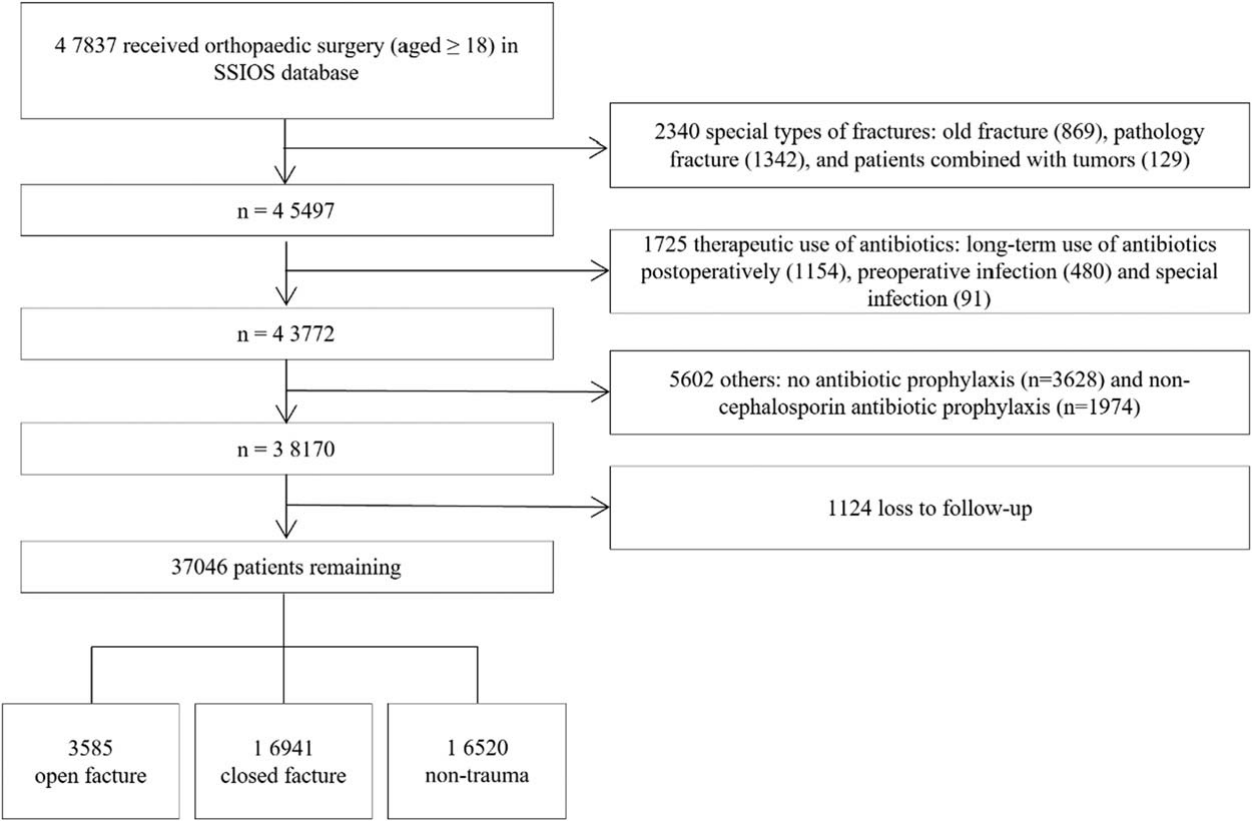
Flowchart showing the patient selection for the study cohort. SSIOS, Surgical Site Infection in Orthopaedic Surgery.

Of the 37 046 patients included (Fig. 1), 55.1% were male, and the mean age was 52.0±15.5 years (range: 18.0–110.0 years); 3585 (9.6%) were open fractures, 16 941 (45.7%) closed fractures and 16 520 (44.6%) non-traumatic surgeries, respectively. The SSI incidence was 2.35% (871/37 046) in the overall cohort, 8.09% (290/3585) for open fractures, 2.56% (433/16 941) for closed fractures, and 0.90% (148/16 520) for the non-traumatic group. Baseline characteristics of the study participants stratified by SAP duration intervals are presented in Supplementary Table S1, Supplemental Digital Content 2, http://links.lww.com/JS9/C94(https://1drv.ms/w/s!AvMSQa964oY8iEVlkaY3rhc29WHg?e=yqTQkB).

### Association between SAP duration and the incidence of SSI

**Figure 2:**
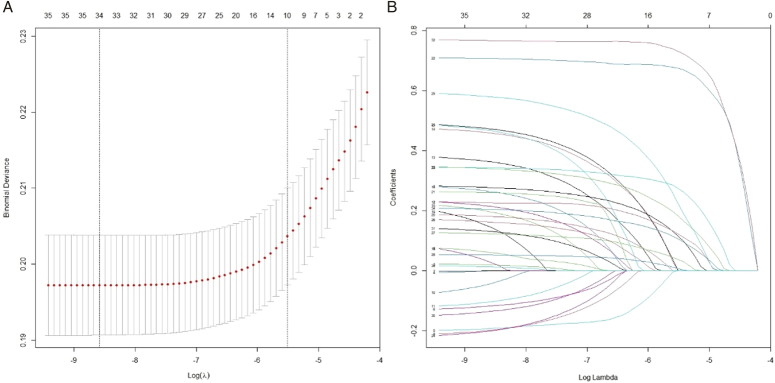
Screening and filtering adjusting covariates for the generalized additive model (GAM) via the least absolute shrinkage and selection operator (LASSO) regression. (A) LASSO coefficient distribution misclassification error. (B) Different colours correspond to various covariates in the distribution of LASSO coefficients for 36 related covariates.

**Figure 3:**
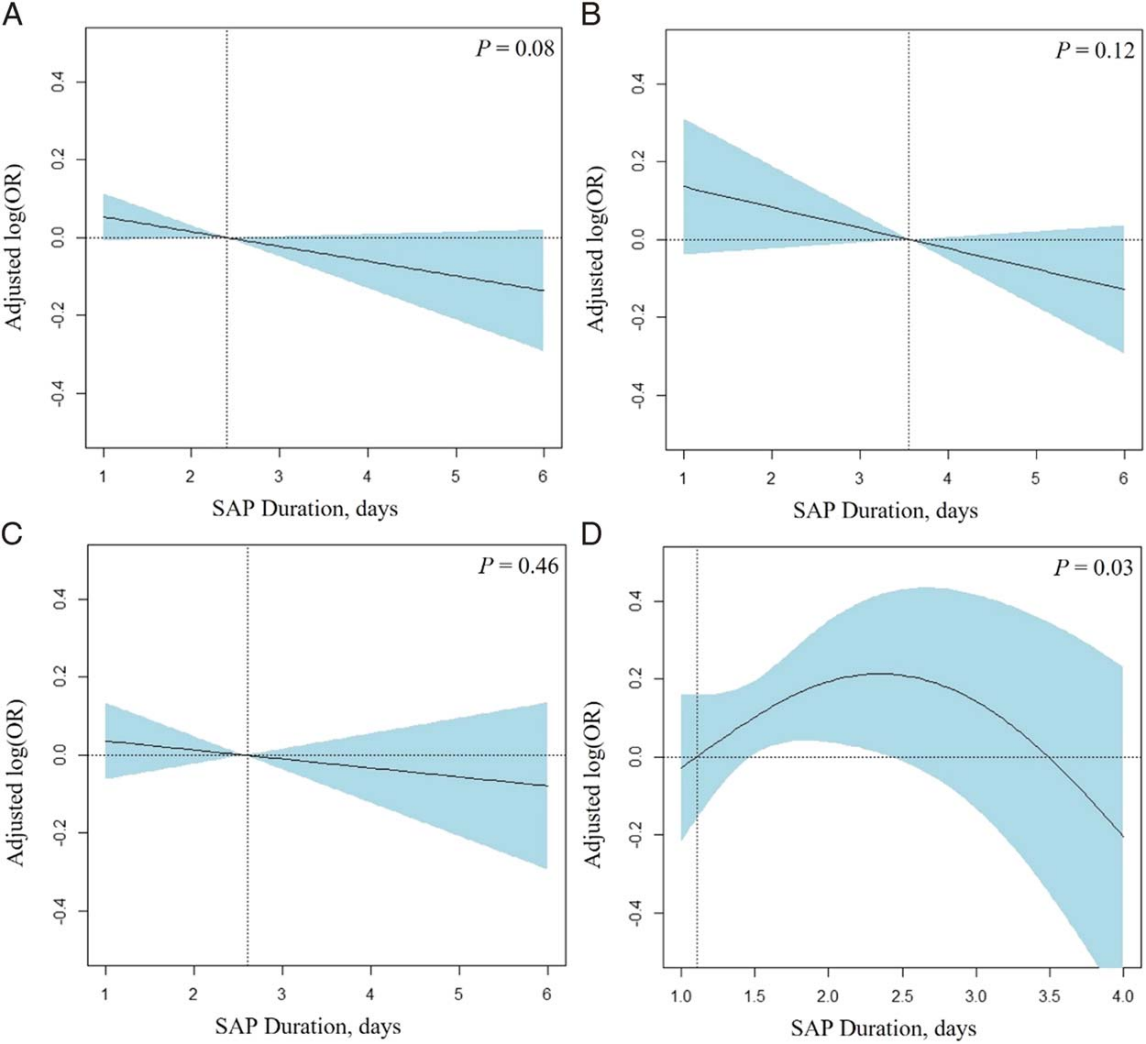
Visualization of the adjusted risk for SSI according to surgical antibiotic prophylaxis (SAP) based on the Generalized Additive Model. Adjusted odds ratios (ORs) for the association between duration of surgical antibiotic prophylaxis and surgical site infection, with *P* values representing the significance of the association. The solid line indicates the OR estimate for surgical site infection; the shaded area, 95% CI; the dashed vertical line, the cut-off value of SAP duration; and the dashed horizontal line, an OR estimate of 1.0. (A) Overall model; (B) Open fracture group; (C) Closed fracture group; (D) Non-traumatic group.

No collinearity was present among variables (VIF <2; Supplementary Table S2, Supplemental Digital Content 2, http://links.lww.com/JS9/C94). LASSO regression analysis identified 34 variables eligible for inclusion for adjustment in GAMs (Fig. 2, Supplementary Table S3, Supplemental Digital Content 2, http://links.lww.com/JS9/C94). No significant relationships of SAP duration with SSI were found for the overall cohort, open fracture, or closed fracture group (*P*>0.050), but a significant non-linear relationship was finding for non-traumatic group (*P*=0.030; Fig. 3). The cut-off points determined by GAMs were 2.4 for the overall cohort, 3.6 for open fracture, 2.6 for closed fracture, and 1.1 for non-traumatic group. In logistic regression model (Table 1), no statistically significant protective effect was observed of prolonged SAP duration against SSI observed in the overall model [adjusted odds ratio (aOR)=0.868; 95% CI, 0.746–1.011] and three groups (open: aOR=0.867; 95% CI, 0.668–1.124; closed: aOR=0.925; 95% CI, 0.754–1.135; non-trauma: aOR=1.184; 95% CI, 0.832–1.683).

**Table 1:** Adjusted logistic regression results for the relationship between SAP duration and the SSI incidence.

	No. (%)		Logistic Regression Model			
Group	≤Cut-off points^a^	>Cut-off points^a^	Unadjusted OR (95% CI)	*P*	Adjusted OR (95% CI)	*P*
Overall	501/24 746 (2.02%)	370/12 300 (3.00%)	1.501 (1.310–1.720)	<0.001	0.868 (0.746–1.011)	0.069
Open fracture	153/1792 (8.54%)	137/1793 (7.64%)	0.886 (0.697–1.127)	0.325	0.867 (0.668–1.124)	0.281
Closed fracture	261/10 361 (2.52%)	172/6580 (2.61%)	1.039 (0.855–1.262)	0.703	0.925 (0.754–1.135)	0.455
Non-trauma	70/7290 (0.96%)	78/9230 (0.85%)	0.879 (0.636–1.216)	0.436	1.184 (0.832–1.683)	0.348

The adjusted covariates included in the overall model and three subgroups were provided in the Supplementary Materials, Supplemental Digital Content 2, http://links.lww.com/JS9/C94 (Supplementary Method https://1drv.ms/w/s!AvMSQa964oY8iEVlkaY3rhc29WHg?e=yqTQkB).

a Cut-off points were 2.4 for the overall cohort, 3.6 for open fracture, 2.6 for closed fracture, and 1.1 for non-traumatic group.

OR, odds ratio; SAP, surgical antibiotic prophylaxis; SSI, surgical site infection

According to the overall adjusted GAM, the SAP duration ranked 21st among the 34 variables (Table 2). The relative contribution ranks of three subgroups ranked 14th (open fracture group), 28th (closed fracture group), and 3rd (non-traumatic group) among the 33 variables, with statistical significance observed only for the non-traumatic group (*P*=0.030). The statistically significant top five factors in the overall cohort were cause of surgery, ALB, wound class, surgical history, and surgery duration. The variables with the greatest contribution in three subgroups were surgery duration (open fracture group), wound class (closed fracture group), and blood transfusion type (non-traumatic group), all of which were statistically significant.

**Table 2:** Relative contribution of model covariates for surgical site infection risk^a^.

	Overall		Open fractures		Closed fractures		Non-trauma	
Variable	*χ* ^2^−*df*	Rank	*χ* ^2^−*df*	Rank	*χ* ^2^−*df*	Rank	*χ* ^2^−*df*	Rank
Overall	450.95		90.4		225.41		62.5	
Sex	0.92	23	−0.87	27	2.66	13	0.73	15
Age	−0.84	34	1.27	15	−0.11	26	−1.17	31
Place of Residence	5.29^b^	16	9.69^b^	6	−0.83	30	0.21	17
Smoking Status	−0.39	30	−1.66	32	1.69	15	2.39	9
Alcohol consumption	−0.32	29	−0.99	28	−0.61	29	1.72	13
Hypertension	0.24	25	−0.43	21	−0.96	31	−0.97	25
Diabetes mellitus	5.64^b^	15	2.61	12	1.10	20	1.52	14
CVD	10.01^b^	11	−0.86	26	11.46^b^	7	0.46	16
CHD	21.21^b^	9	11.15^b^	4	5.47^b^	10	6.57^b^	4
CKD	−0.07	27	0.29	17	−0.99	32	−0.98	26
CLD	3.98^b^	18	−0.84	24	7.05^b^	9	−0.96	24
COPD	1.77	22	0.96	16	−0.28	27	3.77^b^	8
PVD	−0.31	28	−1.00	30	1.14	19	−1.00	28
RBC	8.58^b^	14	0.19	18	5.15^b^	11	−0.28	21
APTT	9.05^b^	13	2.75^b^	11	2.11	14	−0.38	23
WBC	4.08^b^	17	3.76^b^	9	0.57	24	−0.99	27
Serum creatinine	−0.83	32	−1.00	31	1.29	18	0.14	18
ALB	49.88^b^	2	6.97^b^	7	25.13^b^	4	4.08^b^	7
History	37.49^b^	4	1.49	13	32.91^b^	2	6.80^b^	2
ASA	2.22	20	−0.74	22	−2.37	33	−1.00	30
Emergency	−0.83	33	−0.25	19	1.48	17	−1.00	29
Cause of Surgery	107.50^b^	1						
Surgery Location	20.12^b^	10	3.68	10	32.37^b^	3	−2.00	32
Type of Surgery	24.36^b^	7	−0.86	25	0.71	23	4.90^b^	6
Anaesthesia Type	0.03	26	−0.40	20	0.17	25	−0.33	22
Wound Class	43.28^b^	3	11.63^b^	3	46.00^b^	1	5.28^b^	5
Surgery Duration	30.90^b^	5	18.48^b^	1	17.93^b^	5	−2.48	33
Year	9.66^b^	12	−3.41	33	17.23^b^	6	2.29	10
Blood Transfusion Type	28.57^b^	6	14.59^b^	2	4.58	12	24.00^b^	1
Steroid Use	0.39	24	−1.00	29	0.89	22	0.10	19
Antibiotic Choice	24.17^b^	8	10.46^b^	5	10.45^b^	8	2.21	12
Preoperative Dosage	3.95^b^	19	4.09	8	0.98	21	−0.08	20
Postoperative Dosage	−0.76	31	−0.77	23	1.50	16	2.24	11
SAP Duration	2.01	21	1.42	14	−0.46	28	6.71^b^	3

a *χ* ^2^−*df* is the *χ* ^2^ estimate minus the *df* for the variables in the model term.

b Covariate is significant (*P*<0.05).

ALB, albumin; APTT, activated partial thromboplastin time; ASA, American Society of Anaesthesiologists’ score; CHD, coronary heart disease; CKD, chronic kidney disease; CLD, chronic liver disease; COPD, chronic obstructive pulmonary disease; CVD, cerebrovascular disease; PVD, peripheral vascular disease; RBC, red blood cell; SAP Duration, duration of surgical antibiotic prophylaxis; WBC, white blood cell.

### Sensitivity and exploratory analyses

The interaction terms indicate that sex, age, BMI, and CREA have no effect on the primary outcome (Table 3). Our exploratory analysis revealed that patients who exceeded 2 days of SAP were associated with a higher risk of MRSA and MDR infections compared to those who discontinued SAP within 2 days (MRSA: aOR=2.670; 95% CI, 1.689–4.222; MDR: aOR=2.007; 95% CI, 1.480–2.721; Table 4), and a duration-dependent relationship was observed between SAP duration and MRSA and MDR infections in the overall cohort (Supplementary Table S9, Supplemental Digital Content 2, http://links.lww.com/JS9/C94). A similar result was observed in both closed fracture and non-traumatic groups. The main results remained robust in several sensitivity and exploratory analyses (Supplementary Tables S4–S8, Supplemental Digital Content 2, http://links.lww.com/JS9/C94).

**Table 3:** Sensitivity analyses with interaction terms included in the logistic regression model.

	Logistic Regression Model	
Group	Adjusted OR (95% CI)	*P* for Interaction
Overall	0.537 (0.227–1.274)	0.158
Open fractures	0.666 (0.213–2.081)	0.484
Closed fractures	0.937 (0.363–2.422)	0.893
Non-trauma	2.779 (0.592–13.036)	0.195

Cut-off points were 2.4 for the overall cohort, 3.6 for open fracture, 2.6 for closed fracture, and 1.1 for the non-traumatic group.

The following interaction terms were simultaneously included in both the overall model and the three subgroups: SAP Duration×Age, SAP Duration×Sex, SAP Duration×BMI, and SAP Duration×CREA.

CREA, serum creatinine; SAP, surgical antibiotic prophylaxis.

**Table 4:** Exploratory analyses on the association of SAP duration and SSI based on drug susceptibility test.

	Overall		Open fractures		Closed fractures		Non-trauma	
	OR (95% CI)	*P*	OR (95% CI)	*P*	OR (95% CI)	*P*	OR (95% CI)	*P*
MRSA	2.670 (1.689–4.222)	<0.001	0.816 (0.397–1.677)	0.580	4.033 (2.151–7.562)	<0.001	6.989 (1.321–36.970)	0.022
MDR	2.007 (1.480–2.721)	<0.001	0.827 (0.505–1.352)	0.448	3.361 (2.162–5.224)	<0.001	3.796 (1.720–8.378)	0.001
Multiple	1.272 (0.826–1.959)	0.275	0.948 (0.538–1.671)	0.853	2.156 (1.119–4.155)	0.022	2.496 (0.406–15.338)	0.323

Cut-off points were 2.4 for the overall cohort, 3.6 for open fracture, 2.6 for closed fracture, and 1.1 for the non-traumatic group.

The adjusted covariates included in the overall model and three subgroups were provided in the Supplementary Materials, Supplemental Digital Content 2, http://links.lww.com/JS9/C94 (Supplementary Method https://1drv.ms/w/s!AvMSQa964oY8iEVlkaY3rhc29WHg?e=yqTQkB).

MDR, multidrug-resistant; MRSA, methicillin-resistant *Staphylococcus aureus*.

## Discussion

To our knowledge, this prospective cohort study is the first to treat SAP duration as a continuous variable and use GAM models to fit and assess the relationship between SAP duration and SSI, and to quantify the relative contribution of SAP duration among various factors associated with the risk or mitigation of SSI in orthopaedic surgery. We found that prolonging SAP for more than one day after surgery provided no additional protective effects in preventing SSI. The results remained robust in the sensitivity analyses, and reinforced the evidence supporting current guidelines.

The association between SAP duration and the incidence of SSI exhibited distinct patterns across three subgroups. For open and closed fracture groups, there was no significant duration-dependent association between SAP duration and SSI incidence (Fig. 3). Also, the subsequent logistic regression included SAP duration (in days) as the ordered categorical variable produced similar results, with the 95% CI of the OR fluctuating within a narrow range (open fracture: 0.421–1.353; closed fracture: 0.576–1.438; Supplementary Table S4, Supplemental Digital Content 2, http://links.lww.com/JS9/C94). These findings are consistent with previous studies^12,39^ and indicate that the protective effect of SAP reaches a ‘ceiling effect’ within one day after surgery; further prolonging SAP does not provide additional benefits for patients with open and closed fractures. In contrast, prolonging SAP has a non-linear relationship with an increased risk of SSI for the non-traumatic group (Fig. 3). Selective pressure exerted on the skin’s resident microbiota by continuous antibiotic use may disrupt its protective role against opportunistic pathogenic bacteria, consequently increasing the risk of infection. The expansion of the range of SAP regimens in a subset of high-risk patients (e.g. severe diabetes and multiple surgeries) may also play a role. Additionally, our exploratory analysis showed that SAP prolongation was associated with antibiotic-resistant infections in a duration-dependent manner in closed and non-traumatic groups, consistent with findings from several studies (Supplementary Table S9, Supplemental Digital Content 2, http://links.lww.com/JS9/C94)^3,40^. These data demonstrate the daily risk of SAP prolongation on antibiotic resistance, which may be useful for antibiotic stewardship programs by providing a more precise quantification of this risk.

Our results showed SAP duration ranked 14th and 28th in open and closed fracture groups, respectively; and neither had statistical significance. The reasons for the relatively low ranking of duration are likely multifactorial. Intravenous antibiotics can only be delivered to tissues with sufficient blood supply, but this pathway is more likely to be compromised in injured tissues or blood vessels during open and closed fractures. In this instance, antibiotics may fail to achieve their minimum inhibitory concentration (MIC) at the local incision or wound^41,42^, even with prolonged SAP at adequate dosages. Moreover, the significant effects of other factors (e.g. antibiotic choice and preoperative dosage) associated with SSI may dilute the relative contributions of SAP duration. This suggests that prolonging SAP may not be as wise as administering a brief yet adequate dosage with the correct choice of antibiotic preoperatively^43^. For non-traumatic group, SAP duration ranked 3rd among all factors. Unlike open or closed fractures, the absence of trauma-related factors (e.g. injured tissues or blood vessels, contaminated wounds) may make the relative contributions of SAP duration more prominent. However, this does not imply that prolonging SAP provides additional benefits; instead, an increased risk of SSI was observed in the GAM with SAP prolongation (Fig. 3D). Therefore, it is advisable to discontinue SAP within one day postoperatively in patients undergoing non-traumatic surgery. Furthermore, our data showed that the cause of surgery, ALB^44^, wound class, surgical history, and surgery duration^45^ were among the top five factors contributing to the incidence of SSI in the overall cohort. This suggests that prioritizing these factors may be more important than SAP prolongation in comprehensive strategies to prevent SSI.

Our findings that prolonging SAP beyond one day after surgery provides no additional protective effects in preventing SSI but increases the risk of antibiotic resistance infection in some subgroups (closed fracture and non-traumatic group) support the current guideline recommendation that SAP be discontinued within one day postoperatively. These results provide the most up-to-date evidence on the emergence of antibiotic resistance, a concern that has been emphasized in expert opinions but supported by limited data^2,3,40^, and underscores the importance of optimizing SAP duration as a key aspect of antibiotic stewardship to reduce unnecessary antibiotic use and mitigate the development of antibiotic resistance. Based on the data presented, it is theoretically possible to reduce the additional SAP prolongation by 58.5%, consistent with estimates by Karbalaei and Keikha^46^, and thus lead to a reduction in antibiotic-resistant bacterial infections by 12.6–38.2%. However, enhancing adherence to guidelines remains a significant challenge in practice, as current guideline compliance ranges only from 28.5% to 61.3%^47,48^, despite the issuance of the most recent guidelines over 6 years ago^2^. Our other important finding that the contribution of SAP duration ranked among the bottom one-third in the overall cohort and closed subgroups, lower than most common modifiable factors (e.g. ALB, surgery duration, blood transfusion type, antibiotic choice), suggests the need for a re-evaluation of SAP prolongation by both healthcare providers and researchers, and informs them about the role of SAP prolongation in SSI prevention. Notably, this finding is first reported and deviates significantly from prevailing public perception, which may help contribute to improved adherence to guidelines. Sustained antibiotic stewardship efforts are required, along with future work that identifies where these efforts can be most successfully targeted.

Our study has several strengths. First, our data came from a large prospective clinical dataset with long follow-up periods, where patients undergoing orthopaedic surgery were divided into three subgroups for analysis. Second, the GAM used in this study enhanced flexibility in capturing duration-dependent relationships between SAP duration and outcomes, improved the ability to handle high-dimensional data, and allowed for the inclusion of a wider range of covariables. Third, the relative contribution of common factors helps healthcare providers assess the priority of corresponding interventions or preventive measures and allocate medical resources rationally. Furthermore, we conducted sensitivity and subgroup analyses to explore potential heterogeneity.

### Limitations

Our study has several limitations. First, we used bacterial culture positivity as the diagnostic criterion for infection and classified those with infection symptoms/signs but negative bacterial cultures as non-infection cases, which carries the risk of underestimating the incidence of infection. However, given doctors’ fears and concerns about this complication and the routine practice of repeated culture checks at our hospital, the risk of false negatives, while present, was likely minimal. Additionally, the infection rate we reported was consistent with that reported in literatures^10,11^, making it unlikely to substantially affect our primary findings. Second, several factors that might interfere with our outcome, such as preoperative medical and surgical aseptic environment disinfection measures and methods for eliminating bacterial colonization at the surgical site^26^, were not controlled for. We are a specialized orthopaedic hospital with an annual surgical volume of tens of thousands of people, characterized by a high level of standardization and conformity to guidelines; additionally, as a university hospital, there is an objective requirement for orthopaedic physicians to have a higher level of compliance with textbooks and guidelines in clinical practice. These advantages help mitigate the bias introduced by factors that were not controlled for. Third, this study represents a retrospective analysis of data from a prospective single-centre database, which might limit the generalizability of the results. However, compared to multicentre studies, especially those relying solely on administrative databases, our data entry approach is more accurate and precise and encompasses a greater variety of variables. This approach allows us to estimate effect values better. Given that SSI is a rare occurrence, conducting a randomized trial is unlikely. We believe that addressing this important clinical issue can only be achieved through an observational large sample real-world study.

## Conclusion

Prolonged SAP after surgery does not provide a protective effect against SSI in patients who underwent orthopaedic surgeries. Our findings support the current guidelines against the practice of continuing SAP postoperatively. Regarding SAP duration, longer is not better than shorter.

## Ethical approval

Our study obtained approval from the Ethics Committee of the Third Hospital of Hebei Medical University in 2014, with approval number 2014-015-1.

## Consent

As this study relies on secondary analysis of anonymized data, informed consent was deemed unnecessary.

## Source of funding

Hebei Medical University 14th Five-Year Clinical Medicine Innovation Research Team Support Plan.

## Author contribution

Z.Y. and Y.W.: writing the paper and study design; Z.W.: data analysis or interpretation; J.L., P.D., H.M., K.Z., J.Z., M.L., Z.J., Z.P., D.Y., K.D., H.L., J.W., X.X., and Z.S.: data collection; Y.Z. and W.C.: writing the paper and study concept or design; Y.Z.: study concept.

## Conflicts of interest disclosure

The authors declare no conflicts of interest.

## Research registration unique identifying number (UIN)

1. Name of the registry: Chinese Clinical Trial Registry (ChiCTR).
2. Unique identifying number or registration ID: ChiCTR-PPC-14005360.
3. Hyperlink to your specific registration (must be publicly accessible and will be checked): https://www.chictr.org.cn/searchproj.html?title=&officialname=&subjectid=&regstatus=&regno=ChiCTR-PPC-14005360&secondaryid=&applier=&studyleader=&createyear=&sponsor=&secsponsor=&sourceofspends=&studyailment=&studyailmentcode=&studytype=&studystage=&studydesign=&recruitmentstatus=&gender=&agreetosign=&measure=&country=&province=&city=&institution=&institutionlevel=&intercode=&ethicalcommitteesanction=&whetherpublic=&minstudyexecutetime=&maxstudyexecutetime=&btngo=btn.

## Guarantor

Yingze Zhang.

## Data availability statement

Data will be made available from the corresponding author Yanbin Zhu on reasonable request.

## Provenance and peer review

Not invited.

## Supplementary Material
